# Investigating the evolutionary dynamics of diploid number variation in *Ctenomys* (Ctenomyidae, Rodentia)

**DOI:** 10.1590/1678-4685-GMB-2023-0180

**Published:** 2024-02-05

**Authors:** Thays Duarte de Oliveira, Thales R.O. de Freitas

**Affiliations:** 1 Universidade Federal do Rio Grande do Sul, Programa de Pós-Graduação em Biologia Animal, Porto Alegre, RS, Brazil.; 2 Universidade Federal do Rio Grande do Sul, Programa de Pós-Graduação em Genética e Biologia Molecular, Porto Alegre, RS, Brazil.

**Keywords:** Chromosomal rearrangements, karyotype evolution, Rodentia, evolution models

## Abstract

Contrary to predictions from classical hybrid sterility models of chromosomal speciation, some organisms display high rates of karyotype variation. *Ctenomys* are the current mammals with the greatest interspecific and intraspecific chromosomal variation. A large number of species have been studied cytogenetically. The diploid numbers of chromosomes range from 2n = 10 to 2n = 70. Here, we analyzed karyotype evolution in *Ctenomys* using comparative phylogenetic methods. We found a strong phylogenetic signal with chromosome number. This refutes the chromosomal megaevolution model, which proposes the independent accumulation of multiple chromosomal rearrangements in each closely related species. We found that Brownian motion (BM) described the observed characteristic changes more thoroughly than the Ornstein-Uhlenbeck and Early-Burst models. This suggests that the evolution of chromosome numbers occurs by a random walk along phylogenetic clades. However, our data indicate that the BM model alone does not fully characterize the chromosomal evolution of *Ctenomys*.

## Introduction

Chromosome speciation models have been much discussed and criticized by some researchers advocating genetic causes of speciation ( Coyne et al., 1993; Coyne and Orr, 1998). The most widely cited reasons for doubting the critical role of karyotypic changes in speciation include 1) the observation that many chromosomal rearrangements have little effect on fertility ( Sites and Moritz, 1987; Coyne *et al.*, 1993; Dobzhansky, 1933); 2) the theoretical difficulties associated with the fixation of strongly subdominant chromosomal rearrangements in the population ( Walsh, 1982; Lande, 1985; Baker and Bickham, 1986); 3) the alleged ineffectiveness of chromosomal differences as barriers to gene flow ( Barton, 1979; Futuyma and Mayer, 1980); 4) the widespread belief that prezygotic and ecological barriers appear before chromosomal rearrangements in speciation processes and, therefore, are more likely causes of speciation ( Coyne and Orr, 1998; Schemske, 2000).

The concept of karyotypic megaevolution originated from a study conducted by Baker and Bickham in 1980. Their research involved a cladistic analysis of various closely related species but exhibited vastly different rates and forms of chromosomal alterations. Chromosomal rearrangements (CRs) trigger speciation by reducing fertility in chromosomal heterozygotes (when CR is subdominant) or/and by inhibiting recombination (when CR is neutral and does not affect fertility in chromosomal heterozygotes) ( Faria and Navarro, 2010). CR preserves postzygotic isolation between established species and protects hybrid lineages from fusion ( Larkin et al., 2009). CRs protect blocks of linked genes from recombination and are essential for adaptive evolution. Chromosomal fusion and division alter the number of chromosomes and, thus, the number of linkers ( Dumont and Payseur, 2011). Indeed, as part of genome architecture, chromosomal rearrangements are considered an inherently selectable feature ( Hipp, 2007; Avelar et al., 2013).

For the genus *Ctenomys*, there is a classic idea that the chromosomal speciation model is responsible for the appearance of the various species that constitute the genus ( Reig and Kiblisky, 1969; Reig *et al.*, 1990). This idea occurs because *Ctenomys* species meet the expected conditions for this to occur, such as high intra- and interspecific karyotype variation formation of small, isolated populations and low gene flow ( Patton and Sherwood, 1983; Reig, 1989; Freitas, 1995; Malizia et al., 1995). Chromosomal rearrangements in heterozygotes in small isolated populations could generate new karyotypes by genetic drift, which would be tested by selection and low gene flow ( Freitas, 2021).

The diversity of chromosomes in various groups of organisms reveals that numerous lineages display a consistent karyotype among species, particularly with the absence or minimal interspecific variation in chromosome numbers ( Romanenko et al., 2007; Romanenko *et al.*, 2012). This stability accords with the fact that new chromosomal rearrangements are generally associated with heterozygote disadvantage. Therefore, its distribution and probability of fixation in a large population are low ( White, 1978; Coyne and Orr, 1998). The groups of rodents with karyotypes considered more conserved about the ancestor are the species belonging to the suborders Castorimorpha and Anomaluromorpha ( Ward et al., 1991). 

In contrast to this apparent uniformity, several examples of chromosome number diversity in small groups of animals ( Brown et al., 2004) and plants ( Félix and Guerra, 2000) are known. For rodents, species of the suborder Myomorpha have highly reorganized karyotypes ( Graphodatsky et al., 2011) and heterochromatin variations ( Patton and Sherwood, 1982; Svartman et al., 2005; Graphodatsky *et al.*, 2011). The subterranean rodent genera usually present high rates of chromosomal evolution among mammals ( Savić et al., 2017; Li et al., 2020). *Ctenomys* is the group of current mammals with the greatest chromosomal variation. Among species, the diploid number varies from 2n = 10 to 2n = 70 ( Reig et al., 1990). Karyotype variation in *Ctenomys* is determined by the fixation of several chromosomal rearrangements: Robertsonian translocations, pericentric inversions, including insertions or deletions of constitutive heterochromatin ( Reig and Kiblisky, 1969; Cook et al., 1990; Ortells et al., 1990; Giménez et al., 2002; Novello and Villar, 2006; Kubiak et al., 2020). 

The karyotype with the lowest chromosome number is described for *C. steinbachi* with 2n = 10 and FN = 16 ( Anderson et al., 1987). Moreover, the largest 2n = 70 for *C. pearsoni* and *C. dorbignyi*, with different chromosomal formulas (cytotypes), *C. pearsoni* has FN = 80 ( Villar et al., 2014) and *C. dorbignyi*, FN = 84 ( Garcia et al., 2000).

The rhythm and dynamics of this uncontrolled evolution of the number of chromosomes are still little studied, even more so in rodents (see Eichler and Sankoff, 2003; Hipp, 2007; Kandul et al., 2007; Leitch et al., 2010; Lukhtanov et al., 2011; Chung et al., 2012; Vershinina and Lukhtanov, 2013; Lukhtanov, 2014; Lucek, 2018). Since the 1990s, there has been greater interest in the analysis of microevolutionary processes (selection, drift, and mutation) acting on quantitative traits, with a focus on how to obtain estimates of their relative importance from comparative data ( Hansen and Martins, 1996; Smith, 2011; Uyeda and Harmon, 2014). Some statistical models have been proposed to simulate the evolution of quantitative traits, three of which have received the most attention. The first is Brownian motion (BM), which has been used to model evolution by a random process of genetic drift ( Felsenstein 1973). The second is Ornstein-Uhlenbeck (OU), which fits a random walk with a central tendency toward a particular range of phenotypes representing an adaptive optimum ( Cressler et al., 2015). The third is the Early Burst (EB), which initially assumes a rapid evolution followed by a relative stasis ( Harmon *et al.*, 2010). While BM is an evolution-neutral model, OU and EB assume adaptive evolutionary mechanisms.

To better understand the mechanisms of evolution of chromosomal number variability for the genus *Ctenomys*, we used comparative phylogenetic methods to track forms of chromosomal changes during the evolution of this taxon. Thus, we tested the phylogenetic signal of chromosomal alterations in *Ctenomys* by combining phylogenetic data with karyotype information (reviewed in Buschiazzo et al., 2022). We also seek to identify the evolutionary mechanism that best fits chromosomal alterations, testing whether chromosomal alterations evolved in a more neutral way or through adaptive evolution.

## Material and Methods

### Phylogeny reconstruction

Sequences of the cytochrome b gene ( *Cyt-b* - complete CDS: 1146 bp) were collected from GenBank, 46 sequences of *Ctenomys*, and two Octodontidae used as outgroups, all with available diploid numbers ( Table S1). The sequence alignments were performed using MAFFT ( Katoh and Standley, 2013) with default parameter values. AliView ( Larsson, 2014) was used for sequence editing and visualization. Bayesian Analysis inferred the phylogenetic tree in MrBayes 3.2.6. they were implemented in the CIPRES gateway ( Miller et al., 2010; Ronquist et al., 2012). The evolutionary model F81+G was indicated by the jModelTest2 ( Darriba et al., 2012). The analysis was run for at least 10,000,000 generations, sampling trees every 1,000, with 25% of the initial results as burn-in. MEGAX ( Kumar et al., 2018) was used to measure the divergence of the sequences by Neighbor-Joining phylogenetic reconstruction (data not shown), and ML analysis was conducted using RaxML Black Box on the CIPRES gateway ( Stamatakis, 2014).

### Phylogenetic signal and mode of evolution

We used all diploid number data available; in cases of intraspecific chromosomal variations (populations with stable differentiated karyotypes), we used the most repeated diploid number (modal) for phylogenetic comparative analysis ( Table S1). Such cases were found in *Ctenomys pearsoni*, *Ctenomys minutus,* and *Ctenomys lami.* Chromosome numbers were log-transformed before analysis, so we modeled the evolution of chromosome number as a continuous quantitative character evolution, where the frequency of chromosomal fusions and fissions depends on the number of chromosomes ( Hipp, 2007).

To test for a phylogenetic signal of chromosome number onto the Bayesian phylogenies, we calculated two different indices -Blomberg’s K (κ) and Pagel’s lambda (λ) - using the package phytools: phylosig ( Revell, 2012) in R. We tested each index against the null hypothesis of absence of a phylogenetic signal in which case trait values would be randomly distributed along the phylogeny, using 1000 randomization steps. 

We compared the fit between the number of chromosomes with the phylogeny using three different evolutionary models implemented in the package Geiger: fitContinuous ( Harmon et al., 2008) in R: Brownian motion (BM), Ornstein-Uhlenbeck (OU), Early Burst (EB). While BM is a neutral evolution model, OU and EB assume adaptive evolutionary mechanisms ( Felsenstein, 1973; Harmon *et al.*, 2010; Cressler et al., 2015). We fitted each model to all 1000 post-burn-in Bayesian phylograms and compared them using Akaike’s information criterion corrected for finite sample sizes (AICc). 

## Results

### Phylogenetic reconstruction

**Figure 1 -:**
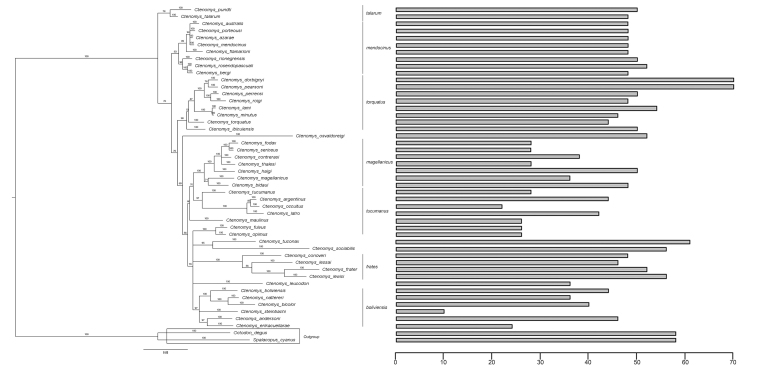
Bayesian majority rule consensus phylogram of the cytochrome b gene ( *Cytb*) for *Ctenomys* species. Nodes supports are shown by posterior probability. Species groups are indicated on the right. The bars indicate the diploid number of each species.

Phylogenetic trees using Neighbor-Joining (NJ), Maximum Likelihood (ML), and Bayesian Inference (BI) methods were obtained for 46 species comprising all the sequence and karyotype data available to date for 65 described species (see Table S1 for the sequence accesses number and diploid number). BI consensus tree topology was the same as NJ and ML tree topology. Figure 1 shows the calculated BI consensus tree, indicating each species’ diploid number. Most nodes were strongly supported; more than half had Bayesian posterior probabilities of 0.90 and higher.

When comparing the phylogenetic groups, the *boliviensis* group presents the most significant karyotype variation between species, from 2n = 10 for *C. steinbachi* to 2n = 46 for *C. boliviensis* and *C. andersoni* ( Figure 1). While the *mendocinus* group shows the least variation ( Figure 1), there are five karyotyped species, four of which have a 2n = 46-48 chromosomes ( *C. australis*, *C. mendocinus*, *C. porteousi*, *C. flamarioni*) and *C. rionegrensis* presents 2n = 52 ( Figure 1).

There are groups of species with identical karyotypes, for example, 2n = 26 in *C. opimus*, *C. fulvus,* and *C. robustus* ( Gallardo, 1979) and 2n = 48, FN = 80 in С. *mendocinus*, and С. roigi ( Ortells, 1995). At the same time, others have the same 2n but different cytotypes, such as *C. haigi*, *C. ibicuiensis,* and *C. yolandae*. All have 2n = 50, but different FNs, 66, 68, and 78, respectively ( Ortells *et al.*, 1990; Gallardo, 1991; Freitas et al., 2012), in addition to the example mentioned above of *C. pearsoni* and *C. dorbignyi* ( Garcia et al., 2000; Villar et al., 2014) *.*

### Phylogenetic signal

**Figure 2 -:**
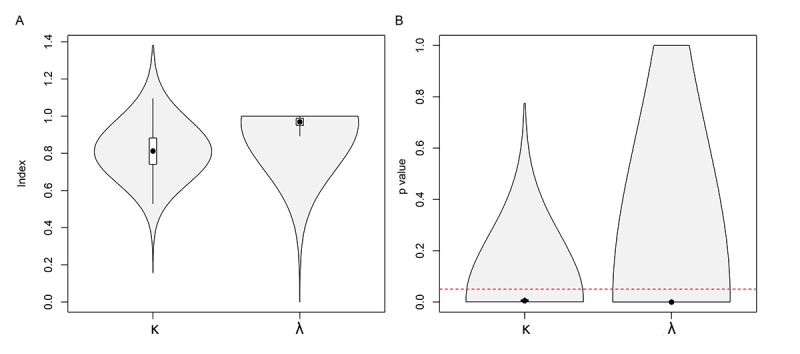
Summary of phylogenetic estimates across 1000 post-burn-in trees. Boxplots depict (A) the observed estimates for Blomberg’s κ and Pagel’s λ with their (B) associated p values. The red line highlights a p-value cut-off of 0.05.

We calculated the phylogenetic signal using Blomberg’s K (κ) and Pagel’s lambda (λ) metrics (Pagel, 1999; Blomberg *et al.*, 2003). As shown in Figure 2 a , κ and λ vary concerning tree topology (including different branching lengths), but for most phylograms, both κ and λ approach the value indicating a strong phylogenetic signal (κ = 0,81, λ = 0.96). Blomberg’s K from κ = 0.15 to κ = 1.37, and Pagel’s lambda ranged from λ = 0.6 to λ = 1. The values obtained for κ and λ were significantly different from those expected by chance (p < 0.05) ( Figure 2 b ).

**Figure 3 -:**
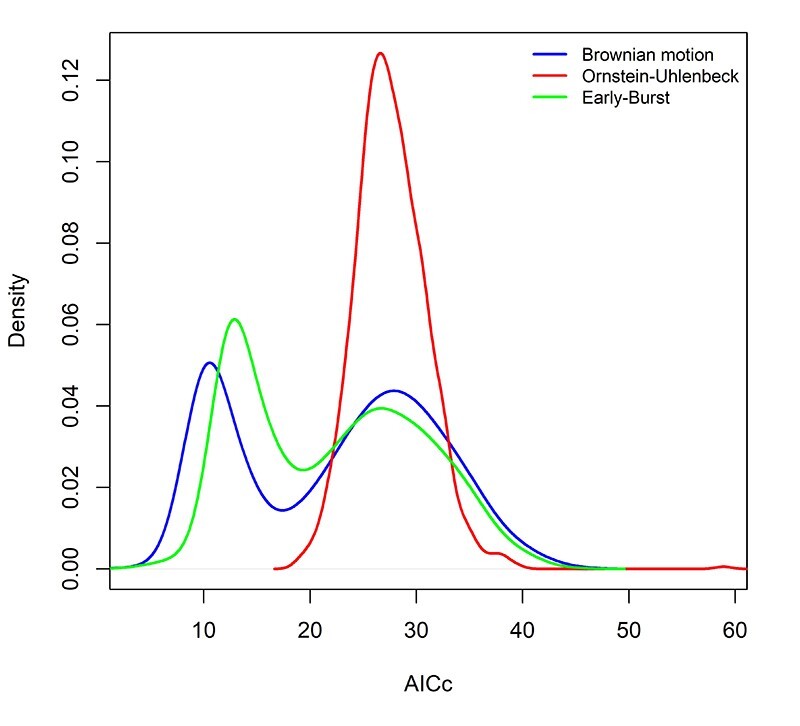
Density distributions for Akaike’s information criterion, corrected for finite sample sizes (AICc) estimated for three different models across the 1000 Bayesian trees: blue - Brownian motion (BM), red - Ornstein-Uhlenbeck (OU), green - Early-Burst (EB).

BM, OU, and EB processes were compared via corrected Akaike information criteria (AICc). Akaike weights demonstrated a higher likelihood rate for the BM model ( Figure 3). Thus, we conclude that the BM model gives a more adequate description of observed trait changes than the OU and EB models. However, the EB model presents values very close to the BM, both better than OU.

## Discussion

Studies correlating genetic and karyotype data still need to be available in animals, especially rodents. Most of these studies were done with butterflies ( Vershinina and Lukhtanov, 2013; Vershinina and Lukhtanov, 2017). Thus, we are testing for the first time a phylogenetic signal and testing evolutionary models, correlating chromosome number variations and the length of phylogenetic branches in *Ctenomys* ( Figure 2). Moreover, we inferred the underlying evolutionary mechanisms by comparing different models of trait evolution ( Figure 3). 

The observed diversity of chromosome numbers in *Ctenomys* could result from multiple CRs that emerged from an ancestral karyotype and accumulated independently in each studied species (reviewed in Buschiazzo et al., 2022). This pattern of chromosomal alteration was described as “karyotype megaevolution”. This model describes a rapid accumulation of multiple CRs occurring independently in each species, which results in a lack of phylogenetic signal ( Baker and Bickham, 1980; Baker and Bickham ,1984). When we see in our phylogeny species from the same group with a significant variation of 2n ( Figure 1), as in the *boliviensis* group, from 2n = 10 for *C. steinbachi* to 2n = 46 for *C. boliviensis* and *C. andersoni*, and this variation is a consequence of fissions and fusions ( Anderson et al., 1987; Cook and Salazar-Ravo, 2004; Gardner et al., 2014), it may seem to correspond to the karyotype megaevolution model. However, our data show a strong phylogenetic signal, the evolution of the number of chromosomes in *Ctenomy*s would be the model of gradual accumulation of similar CRs in sequences of speciation events, which is an alternative to the karyotype megaevolution model ( Lukhtanov et al., 2011; Vershinina and Lukhtanov, 2017).

The results from evolutionary models demonstrated, in most topologies, that BM, but not OU and EB, fits better with the data ( Figure 3). Under a Brownian motion model of trait evolution, this suggests that closely related species are less similar than expected ( Diniz-Filho et al., 2012). This is consistent with the speciation process in *Ctenomys* associated with the allopatric model, as the data showed that each species had a geographical distribution isolated from the others, thus gradually accumulating karyotype changes, resulting in small chromosomal differences between closely related species ( Freitas, 2021). The same has already been reported for butterflies of the genus *Agrodiaetus*, where the Brownian model was better suited because its diversification is an allopatric ( Vershinina and Lukhtanov, 2017).

Our data demonstrated a high phylogenetic signal, but with κ < 1 and λ < 1, indicating that the dynamics of chromosomal evolution in *Ctenomys* follows a different process than just Brownian motion ( Münkemüller et al., 2012; Kamilar and Cooper, 2013). This fact may justify the EB model presenting values very close to the BM, both being better than OU. Thus, the two models (BM and EB) may give a more adequate description of the chromosomal evolution of *Ctenomys*. Consequently, closely related taxa tend to have similar traits. However, we also observed that phylogenetically distant species have similar traits, and some closer species have different traits ( Kamilar and Cooper, 2013). Therefore, our data suggest that chromosomes evolved independently several times during *Ctenomys* radiation ( Figure 1). 

Moreover, CR probably accumulates in *Ctenomys* during a succession of multiple speciation events and results in low and high chromosome numbers. The chromosomal data of GTG-banding and chromosome painting of *Ctenomys* are still incipient; in less than half of the karyotyped species, these techniques were used. Therefore, future studies using GTG-banding and chromosome painting techniques with *C. flamarioni* probes will be extremely important to characterize better and understand CR’s evolution in this taxon.

In *Ctenomys,* other empirical data suggest that chromosomal fusions and fissions are not strongly subdominant and may accumulate gradually ( Freitas, 2021). Thus, our data are hardly compatible with the classic model of chromosomal hybrid sterility; the data demonstrated that chromosomal alterations indirectly or weakly affect the fertility of heterozygotes for CRs. An example is *C. minutus*, a species endemic to southern Brazil, where its populations have notable karyotype variations due to Robertsonian rearrangements, tandem fusions/fissions, paracentric and pericentric inversions, with seven parental karyotypes distributed parapatrically (2n = 50a, 48a, 46a, 42, 46b, 48b, and 50b), among which there is the formation of five intraspecific hybrid zones that give rise to intermediate karyotypes between the parents: 1) 2n = 46a x 2n = 48a → 2n = 47a; 2) 2n = 42 x 2n = 48a → 2n = 43, 44, 45, 46, 47 (5 diploid numbers were found, but 25 different karyotypic combinations); 3) 2n = 46b x 2n = 48b → 2n = 47b; 4) 2n = 50b x 2n = 48b → 2n = 49b; and even the 2n = 49a karyotype, which is possibly a hybrid between 2n = 50a and another karyotype that is still unknown; 5) 2n = 48b x 2n = 42 → 2n = 45b ( Freitas, 1997; Gava and de Freitas, 2002; Freygang et al., 2004; Freitas, 2006; Matzenbacher et al., 2022). Thus, these studies demonstrate that heterozygous *C. minutus* hybrids are fertile.

Therefore, for *Ctenomys,* it demonstrates that the evolution of CR is gradual. Thus, the models of classical theories of chromosomal evolution that generally assume the importance of chromosomal rearrangements as residing in their effectiveness as barriers to gene flow present in the fertility or viability of hybrids may not be the most suitable to explain the process of chromosome evolution from *Ctenomys*. Recent models suggest that, usually, these tests primarily support the notion of gene flow due to a reduction in the recombination rate rather than owing to their impact on fitness ( Noor et al., 2001; Rieseberg, 2001; Navarro and Barton, 2003 a ), which might offer a more accurate perspective. These models are based on: 1) chromosomal rearrangements considered subdominant (translocations, fusions, fissions and inversions) are unpredictable in their effects on allowance, due to interruptions that mitigate or prevent erroneous segregation during meiosis, such as partial or complete deletion recombination ( Coyne et al., 1993); 2) it is extremely difficult to differentiate the effect of chromosomal rearrangements from those of genes on hybrid sterility ( Shaw et al., 1986); 3) the effects of a specific type of rearrangement vary between groups of organisms ( Stebbins, 1958; Sites and Moritz, 1987; Coyne *et al.*, 1993); 4) chromosomal rearrangements often suppress recombination and thus decrease gene flow across genetic regions ( Searle, 1998; Navarro and Barton, 2003a); 5) in some cases a reduction in recombination can result in selection against the recombinant gametes, producing a reduction in the fertility of the hybrids ( Rieseberg, 2001); 6) in chromosomes with characteristic rearrangements, a higher protein evolution rate was identified than in those that did not present this type of alteration ( Navarro and Barton, 2003b).

## Conclusion

In this study, it was possible to test and reinforce the great potential of the genus *Ctenomys* as a model organism for the study of chromosomal evolution, as suggested by Bidau et al. (2003), opening doors for new studies in *Ctenomys*, mainly relating data cytogenetics and phylogenies. We demonstrate the usefulness of the genus *Ctenomys* in studying the role of chromosomal fusion and fission during speciation. Further studies using a wider range of mitochondrial and nuclear genes and genome data, as well as cytogenetic studies, with a particular focus on chromosome painting, are now needed to overcome potential problems associated with observed phylogenetic uncertainties caused by polytomies and assess gene flow’s role in chromosome evolution.

## Supplementary Material

The following online material is available for this article:

Table S1 - Modal karyotype and Cytochrome b sequences of *Ctenomys* used in phylogenetic analyses. Columns from left to right: species, Modal karyotype, GenBank accession number.
