# Supplementary material for: Investigating the evolutionary dynamics of diploid number variation in Ctenomys (Ctenomyidae, Rodentia)
Source: Genet Mol Biol. 2024 Feb 5;46(3 Suppl 1):e20230180. doi: 10.1590/1678-4685-GMB-2023-0180 (PMC10842476; doi:10.1590/1678-4685-GMB-2023-0180)
Supplement: Table S1 - [file 1415-4757-GMB-46-03-s1-e20230180-s1.pdf]

Genetics and Molecular Biology
https://doi.org/10.1590/1678-4685-GMB-2023-0180

Supplementary Material to "Investigating the Evolutionary Dynamics of Diploid Number Variation in *Ctenomys* (Ctenomyidae, Rodentia)"

Table S1 - Modal karyotype and Cytochrome b sequences of *Ctenomys* used in phylogenetic analyses. Columns from left to right: species, Modal karyotype, GenBank accession number.

Species	Modal 2n	Accession number
Ctenomys andersoni	46	AF007047
Ctenomys argentinus	44	AF370680
Ctenomys australis	48	AF370697
Ctenomys azarae	48	MZ333085
Ctenomys bergi	48	OP422502
Ctenomys bicolor	40	JX880049
Ctenomys bidaui	48	OK839692
Ctenomys boliviensis	44	AF007037
Ctenomys conoveri	48	AF007055
Ctenomys contrerasi	38	OK839699
Ctenomys dorbignyi	70	JQ389030
Ctenomys erikacuellarae	24	KJ778555
Ctenomys flamarioni	48	AF119107
Ctenomys fodax	28	HM777475
Ctenomys frater	52	AF007045
Ctenomys fulvus	26	AF370688
Ctenomys haigi	50	HM777476
Ctenomys ibicuiensis	50	JQ389020
Ctenomys lami	54	HM777477
Ctenomys latro	42	HM777478
Ctenomys lessai	46	AF007048
Ctenomys leucodon	36	AF007056
Ctenomys lewisi	56	AF007049
Ctenomys magellanicus	36	HM777479
Ctenomys maulinus	26	AF370703

**Species**	**Modal 2n**	**Accession number**
Ctenomys mendocinus	48	HM777480
Ctenomys minutus	46	HM777481
Ctenomys nattereri	36	HM777484
Ctenomys occultus	22	HM777485
Ctenomys opimus	26	AF007041
Ctenomys osvaldoreigi	52	OP390083
Ctenomys pearsoni	70	HM777486
Ctenomys perrensi	50	HM777487
Ctenomys porteousi	48	AF370682
Ctenomys pundti	50	HM777490
Ctenomys rionegrensis	50	AF119114
Ctenomys roigi	48	HM777492
Ctenomys rosendopascuali	52	OP422504
Ctenomys sericeus	28	HM777496
Ctenomys sociabilis	56	HM777495
Ctenomys steinbachi	10	AF007043
Ctenomys talarum	48	HM777497
Ctenomys thalesi	28	OK839703
Ctenomys torquatus	44	EF372287
Ctenomys tuconax	61	AF370693
Ctenomys tucumanus	28	HM777499
Octodon degus	58	AF007058
Spalacopus cyanus	58	AF007061
